# Practitioners’ perspectives on unintended effects of illicit drug use prevention public service announcements in Australia

**DOI:** 10.1093/heapro/daae185

**Published:** 2024-12-06

**Authors:** Kirsteen Munro, Svetlana Bogomolova, Lucy Simmonds

**Affiliations:** Centre for Social Impact, College of Business, Government and Law, Flinders University, Sturt Road, Bedford Park, South Australia 5042, Australia; Centre for Social Impact, College of Business, Government and Law, Flinders University, Sturt Road, Bedford Park, South Australia 5042, Australia; Centre for Social Impact, College of Business, Government and Law, Flinders University, Sturt Road, Bedford Park, South Australia 5042, Australia

**Keywords:** public service announcements, PSA, illicit drug use prevention, practitioner perspectives, unintended effects, advertising, qualitative methods

## Abstract

Public service announcements (PSAs) or campaigns aimed at preventing harm can inadvertently risk creating additional harms. It remains unclear whether these unintended effects are considered during campaign development, if risk mitigation strategies are implemented, or how professionals involved perceive these issues. It is in the context of illicit drug use prevention PSAs that our research investigates and explores the perspectives of practitioners—health support professionals and advertising campaign designers and creators. Semi-structured expert interviews were conducted to capture and synthesize practitioners’ perspectives which were then analysed by applying a framework to address the unintended effects of public health interventions. The results indicated that practitioners from both sectors are aware of unintended harms but place varying levels of importance on different aspects. In the case of illicit drug prevention PSAs, incorporating practitioners’ perspectives in campaign development may result in mitigating the risk of potential unintended harmful effects.

**Contribution to Health Promotion**

- Illicit drug use prevention campaigns are often deemed ineffective, and even harmful, as the messages neglect the unintended consequences on current consumers and spillover to unintended audiences.
- Health support professionals believe that harmful unintended effects must be mitigated.
- Advertising professionals acknowledge there may be unintended effects but are generally more focused on message reach.
- A broader range of stakeholders should be consulted prior to the design of public service campaigns with the objective of illicit drug use prevention.
- A conceptual framework has been tested to provide guidance when considering the potential harms of illicit drug use prevention PSAs.

## BACKGROUND

It is widely recognized that the use of illicit substances is responsible for many forms of direct and indirect harm (Catford, 2001; United Nations Office on Drugs and Crime and World Health Organization, 2018). Illicit in this context refers to those substances that are illegal in many international jurisdictions, for example, amphetamines, ecstasy, cocaine and heroin. Public service announcements (PSAs) aimed at preventing illicit drug use are a common and visible strategy used by organizations to address and reduce the harms associated with drug use. Two reported positive outcomes of such PSAs are increased efforts to seek further information and discussions with others about issues related to illicit drug use (Stancombe Research and Planning, 2018). However, previous studies indicate that illicit drug use prevention PSAs may also be considered ineffective (Botvin and Griffin, 2016) and have limited impact in influencing the desired behaviour change (Werb *et al*., 2011). Moreover, such campaigns can also have unintended and detrimental effects which could cause harm to both the intended and unintended audiences (Botvin and Griffin, 2016; Ferri *et al*., 2013). Examples of these harms include substantial increases in both the actual use of illicit drugs and individuals’ intention to use them (Ferri *et al*., 2013). It is, therefore, important to consider the factors contributing to campaign (in)effectiveness, and, ultimately, how the process of campaign development can be improved, with a particular focus on considering possible unintended negative consequences.

Typically, illicit drug use prevention PSAs conducted by public health organizations aim to target young people aged between 10 and 24, such as the ‘*Australian National Drugs Campaign 2017*’ (Australian Government Department of Health and Aged Care, 2017), ‘*Just Say No Campaign*’ (tru0091, 2007) and ‘*This is your brain on drugs’* (Kalamut, 2010). This target audience is considered especially vulnerable and susceptible to initiating the use of illicit drugs due to their propensity for risky behaviours (Australian Government Department of Health and Aged Care, 2017). To deter experimentation with illicit drugs, practitioners commonly employ scare tactics in their PSAs (Lancaster *et al*., 2017). A seemingly forgotten segment of the unintended audience of illicit drug use prevention PSAs are those who currently use illicit drugs, and for whom such tactics could be counter-productive, if not harmful—this is elaborated further in the paper. Given the typical nature of mass media used to deliver most illicit drug use prevention PSAs, it is very likely that both groups, intended and unintended, will receive the campaign message. This can result in a ‘spillover’ effect, something that occurs when the action of a marketer impacts the intended or unintended audience in an unintended manner (Desiraju and Van Tran, 2014). It is, therefore, critical for the creation of effective advertising to ensure campaign developers have carefully considered the potential effects of the message on both audiences and that any unintended effects could be negative.

### Intentions and effectiveness of illicit drug use prevention PSAs

Well-designed mass media advertising has the potential to reach large audiences repeatedly over extended periods (Wakefield *et al*., 2010). These campaigns aim to reduce drug use, prevent initiation and encourage cessation (Becker-Olsen and Briones, 2009; Lang and Yegiyan, 2008). While these objectives are often well-intentioned (Stead *et al*., 2019), the effectiveness of the message’s delivery mechanism requires closer examination. Despite varying evaluations of illicit drug use prevention PSAs (Allara *et al*., 2015; Stead *et al*., 2019), one common theme is apparent—there is limited empirical evidence supporting their success (Ferri *et al*., 2013; Schoenbachler and Whittler, 1996; Werb *et al*., 2011). This underscores the need for enhanced evaluation practices that incorporate insights from a wider range of stakeholders. Current evaluations of illicit drug use prevention campaigns largely fail to capture the perspectives of experts from two crucial areas: (i) health professionals who provide specialist support to people who use drugs and (ii) advertising professionals who design and create campaigns, leading to a lack of understanding about how to minimize unintended effects, and whether opportunities exist to address these issues during campaign development.

### Unintended effects of illicit drug use prevention PSAs

Illicit drug use prevention campaign messages are often designed with specific appeals to elicit certain emotions in an attempt to shock, scare and/or threaten receivers of the messages into action (Lupton, 2014; Schoenbachler and Whittler, 1996). For example, fear is a common approach employed by message creators in an effort to discourage the initial and ongoing use of methamphetamine (Lancaster *et al*., 2017). Consequently, those who partake in the use of methamphetamine are often portrayed as dangerous, dishonest, dirty (Erceg-Hurn, 2008) and a threat to the community (Douglass *et al*., 2017). These depictions can potentially intensify both internalized stigma and stigma within the broader community (Douglass *et al*., 2017; Treloar *et al*., 2021). Moreover, certain types of campaigns have been found to increase intentions to use drugs (Lang and Yegiyan, 2008), exacerbate marginalization (Lupton, 2014) and stimulate curiosity leading to experimentation (Crano *et al*., 2013), particularly among specific populations.

### Conceptual framework

The authors did not identify any existing framework specifically designed to address the unintended effects of illicit drug use prevention PSAs. However, initial steps in this direction have been taken by Lorenc and Oliver (2014) and Allen-Scott *et al*. (2014) through their analyses of public health interventions. Our study will adopt the conceptual framework proposed by Lorenc and Oliver (2014) as the five identified harms are more aligned with illicit drug use prevention PSAs. The five types of harms presented in this framework—direct, psychological, equity, group and social and opportunity cost—will be specifically applied to illicit drug use prevention PSAs.

### Research questions

This study seeks to understand whether potential unintended harms of illicit drug use prevention PSAs are considered by two groups of practitioners: (i) health professionals who provide specialist support to people who use drugs and (ii) advertising professionals responsible for the design of the campaigns. We will do so by collecting, analysing and synthesizing the perspectives of these two groups of professionals. The research questions guiding this study are:

1. What are the perspectives of health support practitioners on the unintended effects of illicit drug use prevention PSAs?
2. What are the perspectives of advertising practitioners on the unintended effects of illicit drug use prevention PSAs?
3. How do the perspectives of health support and advertising practitioners align with the adverse effects outlined in a conceptual public health intervention framework (Lorenc and Oliver, 2014)?

## METHODS

### Study setting

The method employed to collect our primary data involved the conduct of semi-structured interviews guided by open-ended questions, enabling in-depth and detailed examination of extensive issues (Anderson, 2010; Neale *et al*., 2005). This method was also conducive to the broadening of and/or deviation from the original scope when new or unexpected information was revealed (Anderson, 2010; Bryman, 2016). Additionally, semi-structured interviews were able to underpin the discovery of patterns with reasons (Busetto *et al*., 2020), relevance and importance (to the interviewee) (Bryman, 2016).

Underpinned by the authors’ use of a subjectivist interpretivism philosophical position, this research was designed to collect, understand and interpret perspectives from participants’ viewpoints (Saunders *et al*., 2019). This philosophical position acknowledges that humans create meaning, which allowed the authors to look at and contextualize the perspectives of different individuals from distinct industry sectors. The authors adopted an empathetic position (Saunders *et al*., 2015), crucial for understanding the participants’ point of view, especially given the tendency for the topic to elicit contrasting perspectives. Ethics approval was granted by Flinders University Human Ethics (Project No. 5230) and the data were collected between May and September 2022.

### Sampling and recruitment

Recruitment was undertaken by way of purposive sampling, a common approach in qualitative research (Anderson, 2010). Health professionals with expertise in supporting people who use drugs, as well as those employed in the advertising sector, were intentionally approached due to their relevant expertise. The sample was recruited by a combination of methods:

(1) drawing from a network of known contacts,
(2) contacting specific organizations following desk research and
(3) contacting individuals who made submissions to an Australian Parliamentary Joint Committee on Law Enforcement Inquiry (Parliament of Australia 2020).

### Participants

Participants represented two sectors—those experienced in providing specialist support to people who use drugs (health support cohort) and those situated in the advertising media sector (advertising cohort). Nineteen participants agreed to be interviewed—12 participants from the health support sector and seven from the advertising media industry. As data saturation (the point at which there is a reliable lack of variation and emergent themes (Bryman, 2016)) was becoming evident, this number of interviews was considered appropriate. The sample size was driven by the interviewing, ongoing analysis and reflection processes and is supported by previous work undertaken with similar industries (e.g. Drumwright and Murphy, 2009).

### Data collection and ethical considerations

Interviews were conducted by the authors and conversations were guided using semi-structured schedules to allow participants to express their own perspectives of experiences; especially eliciting unintended effects of illicit drug use prevention PSAs. A choice of face-to-face in-person or video conferencing via Microsoft Teams or via telephone was offered. 19 interviews in total were conducted—16 via video conferencing using Microsoft Teams and digitally video-recorded; two by telephone, audio-recorded; and one in-person, also audio-recorded. Participants were physically located in Australia, and the interviews lasted between 30 and 90 min. Field notes were taken during and after all interviews. Prior to each interview, all participants received an introductory email and a letter with information about the study (i.e. that participation was voluntary, interviews would be recorded and how their participation would contribute to knowledge, etc.). Participants were assured of complete confidentiality and anonymity of their responses—both in terms of their personal and organizational identities. Only generalized themes and anonymous quotes are used for this publication. Written consent was obtained before each interview commenced and all participants were given the opportunity to review and amend transcribed data and/or watch the recorded interview. One participant opted to review and edit responses.

The interview guides differed slightly for each sector as is evidenced in Supplementary Appendix A. The health support participants were asked about their perceptions of the effects illicit drug use prevention campaigns had on their clientele. The advertising interviewees were asked to consider what effects behaviour-change campaigns might entail and what was taken into consideration at the design and creation stage in relation to unintended effects. Further information was explored when deemed appropriate.

### Data analysis and structure of results

**Fig. 1:**
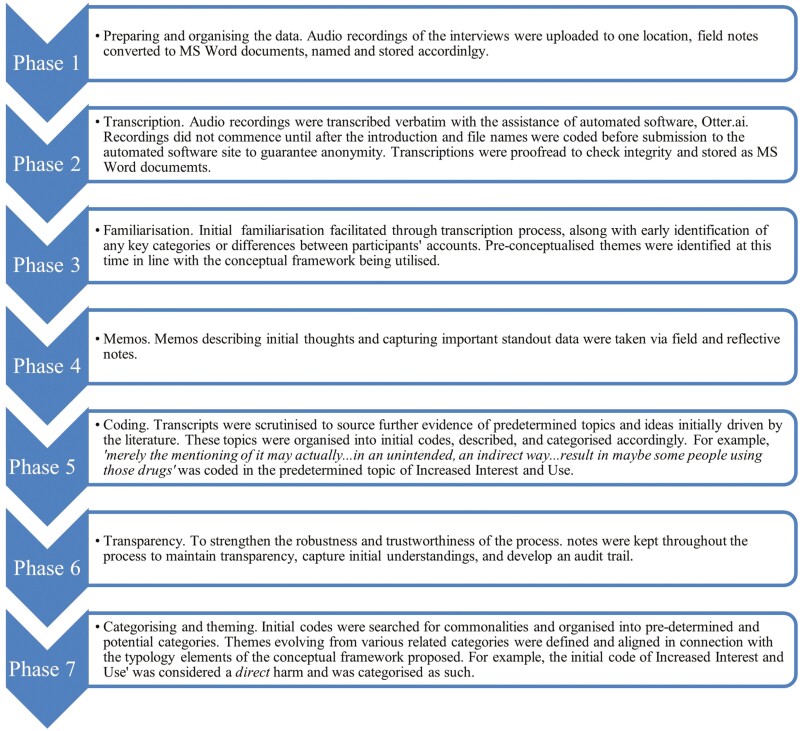
Phases of thematic analysis.

An initial analysis of data was undertaken following two to three interviews from each sector to understand perspectives and the appropriate analytical framework to use. As there were common categories, both within and between cohorts, thematic analysis, a common (Bryman, 2016), independent and reliable method used for analysing interview data (Vaismoradi *et al*., 2013) was employed. Accordingly, the authors followed the seven phases proposed by Lester *et al*. (2020), detailed in Figure 1. The results of the primary data collection thematic analysis are covered under the themes of the adverse effects of public health interventions conceptual framework (Lorenc and Oliver, 2014).

## RESULTS

The first two research questions are concerned with the perspectives of health support and advertising professionals. To answer these questions, perspectives of practitioners from the health support and advertising sectors are thematically presented under each category proposed by Lorenc and Oliver’s (2014) conceptual framework.

### Perspectives of health support and advertising cohorts

Counsellors and social workers who provide specialist support to people who use drugs, along with representatives from drug education and support peak body organizations formed the cohort of health support participants (*n* = 12). In this context, a peak body is a representative, non-government organization advocating for their specific community sector (Ogle and Bowling, 2011). Participants interviewed from the advertising cohort (those who design and create behaviour-change campaigns) included advertising executives, intermediaries, managing directors and general managers experienced in the design, creation and dissemination of either illicit drug use prevention and/or broader behaviour-change advertising (*n* = 7). They represented local, national and international advertising media agencies.

#### Intentions and effectiveness of illicit drug use prevention PSAs

Most health support participants perceived the goals and aims of illicit drug use prevention PSAs as being to stop or deter illicit drug use by (particularly) young people, as well as increase awareness and reduce associated harms through education. However, one participant took a rather cynical view:

*just to get them* [the general community] *to hyperventilate a bit about it, want to lock people up … get them out of our backyard, build up a sense of crisis around individuals that often have multiple layers of complexity in their life... sometimes it’s just virtue-signalling that they* [the government] *care about the affected community.* (SS#6)

The overwhelming opinion was that the campaigns were *not* effective. Most health support participants believed that shock and scare tactics did not achieve the desired outcome or change behaviour and could be damaging. Further, if the target audience is not exposed to the advertisement or is marginalized, an advertising campaign could be especially ineffective.

The role of professional communicators, as defined by the advertising cohort, is to take complex information and distil it. This is achieved by understanding the problem, working out what they need people to know, and what they want people to do as a result. Some of the main aims and goals of illicit drug use prevention PSAs were perceived as increasing target group awareness, decreasing use, preventing and discouraging initial uptake, educating, facilitating long-term behaviour change and increasing people’s understanding of drug addiction. These campaigns should be able to convey details of valuable tools available:

*…all about tapping into the sense that there is actually a way to claw your way back, if you find yourself spiralling and losing control. It was all about how you can find your way back. There is help and there are tools to help you.* (AD#4)

Most of the advertising cohort indicated that effectiveness was primarily measured by message reach and cost-effectiveness. That is, whether the message reached the intended audience and whether the campaign was value for money. These evaluations are typically undertaken in conjunction with, or solely by, the commissioning agency, usually a government body.

*…So how do we get it to the right audience with the right message … from our point of view, we’re looking at this through a lens of advertising effectiveness … did it reach the intended target?* (AD#3)

#### Unintended indirect effects

All health support practitioners identified the existence of unintended effects that can potentially create real and serious direct and indirect harms. The advertising participants acknowledged their awareness of unintended consequences and indicated that care was needed to connect with an audience in an authentic way. However, one agency had a slightly nuanced perspective:

*We’ve been doing this for a really long time, so I think in terms of unintended consequences, we usually know pretty early on what those pain points are likely to be … then it’s, I guess, a matter of in the development of the campaign, how you address it, or if you choose not to.* (AD#2)

In general, the advertising cohort relies on their client, typically a commissioning arm of a government agency, to let them know what messaging might be problematic and then decide how or whether to mitigate any negative effects. Another aspect raised was that the consideration of unintended effects was outside their scope:

*…our role is to make sure whatever the message the department wants to deliver, just reaches that audience.* (AD#3)

Further perspectives of both cohorts are reported in themes under the categories of harms identified by Lorence and Oliver (2014).

### Direct harms

#### Normalization, increased interest and increased drug use

Of concern to the health support participants were the effects the advertising may have on increasing, even initiating, the use of illicit drugs. Some of the benefits of drug use may be unintentionally portrayed, or the fact that a particular drug is mentioned at all can result in increased or initial use:

*…there’s some evidence to say that there are individuals who never would have thought about using any drug who potentially it now brings it to the forefront of their mind. So… merely the mentioning of it may actually… in an unintended, an indirect way… result in maybe some people using those drugs.* (SS#4)

For the advertising cohort, despite the existence of literature suggesting that the normalization of drug use behaviour increases interest in drug use, only two participants alluded to this. One participant found it intolerable, while for the other, it was just a consideration.

*…a risk might be it increases interest in illicit drug use where it normalises the drug use…now that, obviously, is a key risk…we couldn’t tolerate. So, if that was thought to happen, we’re not going to…be participating in that.* (AD#7)

One advertising participant touched on the need to be careful if showing the product in the advertising as it may trigger use, confirmed by other perceptions that if something new is mentioned in the media, young people will want to try it; it prompts interest in use and…

*you’ve done the absolute opposite of what you intended to do … so that would be probably the most awful public health outcome.* (AD#7)

#### Lack of credibility

Most health support participants agreed that advertisements exampling the extreme behaviours of the minority do not resonate with the target audience and are readily dismissed. For example, portraying someone under the influence of methamphetamine exhibiting violent behaviour will be disregarded as most people who take this drug don’t become violent. Similarly, if messages are unrecognisable as the target audience’s own experiences, they are disregarded.

*Under the influence of methamphetamine that* [smashing windows in a hospital] *can happen, but at the same time, most, a lot of people on methamphetamine don’t get violent.* (SS#2)

*I think most people would say, ‘well that’s not me’ … The ads tend to show the extreme, the minority.* (SS#2)

Some young people have a distrust in government, therefore governmental messaging is hard for them to accept (Farrugia, 2014; Farrugia and Fraser, 2017; McEwen and Hanneman, 1972). Additionally, some government advertising has been so far off the mark that it has been ridiculed and considered a joke, therefore lacking any credibility and again, disregarded. The *Stoner Sloth* (aa4398743873, 2015) campaign, a highly criticised Australian anti-cannabis campaign that portrayed users as sluggish, human-sized sloths unable to accomplish simple tasks, was exampled more than once:

*…if I was a teenager in high school, I would just be laughing … I’d be smoking weed with my friends, ‘oh, I’m a sloth.’ You know, it would become a joke.* (SS#5)

Younger audiences were identified by some advertising participants as being sceptical of illicit drug use prevention advertising. If the portrayals and stereotyping of certain populations are not realistic, the communication won’t resonate with the target audience, and they may disconnect from and disregard the message; they don’t think it applies to them as they don’t know anyone who has experienced it and/or it never happens:

*One of the most important things is that everything must feel credible. Stereotyping can be a trap that allows people to exclude themselves from thinking the message has relevance for them personally.* (AD#4)

*…it’s true in relation to behaviour change marketing that, the more you do something without negative consequence, the less likely you are to believe that anything bad will happen to you.* (AD#4)

### Psychological, equity and group and social harms

#### Stigma and marginalization

Stigma implied through illicit drug use prevention PSAs was a major concern to the health support cohort of participants. In their view, those who use illicit drugs are labelled, judged, blamed and isolated by wider society with the resultant stigma reducing the likelihood of seeking help.

*…if you stigmatise a particular group, then they get resistant to the objective of what you’re seeking to pursue … you stigmatise drug users because of the drug seeking habits, you will definitely fail to get them into health seeking behaviours.* (SS#6)

*And what a barrier that then becomes to that person getting better and getting treatment and helping themselves and being able to ask for the help they need.* (SS#5)

The health support cohort also identified that younger people may avoid attending their local general practitioner (GP) out of fear of details of their drug use being passed on to a family member. Attending an alternative GP, combined with the absence of a full medical history, can result in the appropriate care not being received. Those living in smaller communities may be especially susceptible For example, the attendance at an emergency department of a local hospital with a history of drug use may prevent them from receiving appropriate non-related future healthcare.

*So the stigma…attached to go into a family GP is a big one.* (SS#2)

*the healthcare system, I think, unfortunately, the medical profession, by and large, is quite stigmatising towards illicit substance use, I think.* (SS#5)

The health support cohort wanted to see people educated about the language around drug use and people who use drugs as a starting point. They believed some of the language used in advertising is stigmatizing, such as the words ‘*addict*’ and ‘*ice*’, (as in the Australian government’s media campaign *Ice Destroys Lives)* (BCM Group, 2016). Such language is taken on by people who use drugs and is immensely damaging, perpetuating internal stigma.

*And the word addict comes to mind, which is stigmatising and labelling. It’s not person-centred, it’s really about … society looks down upon you. You are something that we are not…a lot of shame attached to it – shame and blame and labelling.* (SS#1)

Three key health support participants had a concern that ineffective advertising resulted in further marginalization of groups of people who use drugs that were already disconnected from society, effectively driving them further away:

*If you’re someone … living, rough-sleeping, using heroin every day, you look around to the rest of the world and you think ‘I’m not a part of this, I don’t belong to any of this…there is a world over there, and there’s me over here.* (SS#5)

Stigma was recognized by all advertising participants, however, only one noted that they actively refrain from using fear and scare tactics as they are less effective with a stigmatized audience. Others recognized that stigma was one of the challenges and a part of the consideration, and importantly, one participant acknowledged that stigma may also affect the *unintended* audience.

*They’re so focused on their target audience, which is preventing usage in that group, they’re not thinking about all the other people here who are like, ‘Oh, hang on, but what about me? I already use this so do you not care? I’m feeling worse’.* (AD#1)

Some participants in the advertising cohort also believe that stigmatizing the target audience can create further damage by the generation of help- and support-seeking barriers.

*…that can be very difficult for already vulnerable groups seeing greater differences between those groups and social exclusion, and then a lack of people feeling comfortable to be able to speak to other people to seek help. Whether that might be in a professional sense, or to speak to family and friends about these things.* (AD#1)

Interestingly, one participant commented that stigma was not necessarily a bad thing as the intention is for people to realise drug addiction is a serious issue:

*It sounds brutal, but you know, our intention was that we want people to realise that this is a serious issue, and to go and talk to somebody about it. So if that means that there’s a stigma about [], then I think that would be a good thing. Whether or not that I mean, that is good for the individual, I don’t know, but as a culture, and … part of the attraction of this campaign was that you want people to go, ‘No, I don’t want to take [].’* … *So I think that stigma around the drug use, I think, is a is a good thing.* (AD#2)

### Opportunity cost harms

Opportunity cost harms arise when funds used to employ a particular intervention that may be ineffective could have been used to make a material difference in another area (Lorenc and Oliver, 2014). Although most health support participants identified barriers to help- and treatment-seeking, such as waitlists, capacity and lack of facilities, it was not directly linked to consideration of redirecting advertising funds towards such resources. No advertising professionals broached the subject.

The third research question was concerned with how practitioners’ viewpoints aligned with a conceptual framework proposed by Lorenc and Oliver (2014) regarding the adverse effects of public health interventions. The perspectives obtained from the practitioners perceived these adverse effects similarly to how they are outlined in the framework in four out of the five categories—direct, psychological, equity and group and social harms, with opportunity cost harms alluded to by health support professionals only. These are now discussed.

## DISCUSSION

Illicit drug use remains a pressing global health issue, prompting widespread efforts to combat it through PSAs aimed at promoting abstinence, reduction and cessation. However, these PSAs can sometimes produce unintended and adverse effects. This study addresses a critical gap in the existing literature by exploring the perspectives of practitioners from two distinct industry sectors who are not currently represented. These professionals—those experienced in supporting individuals with substance use issues and those involved in designing illicit drug use prevention PSAs—hold valuable insights that could help mitigate these negative effects. By integrating their viewpoints, this study not only provides a comprehensive understanding of the challenges associated with illicit drug use prevention PSAs but also applies these insights to a conceptual framework designed to provide guidance when considering the adverse effects of public health interventions.

The findings of this study reveal that health support professionals are generally well-informed about the potential unintended effects of illicit drug use prevention PSAs. While some advertising professionals possess a broad awareness of these potential effects, it is often not their primary focus, with the responsibility of addressing such issues typically falling to the departments commissioning the campaigns. This study offers two significant contributions to theory and literature. First, it begins to address a notable gap by incorporating practitioners’ perspectives on the unintended effects of illicit drug use prevention PSAs, thus enriching the existing body of knowledge. Second, it is the first to apply these practitioners’ insights and relevant literature to the five adverse effect concepts proposed by Lorenc and Oliver (2014). This application not only strengthens the theoretical framework but also provides a deeper understanding of how these effects manifest in real-world contexts.

### Direct harms

Public health interventions can potentially increase the risk of physical injury or harm (Lorenc and Oliver, 2014). When illicit drug use prevention PSAs are ineffective in influencing desired behaviours such as cessation, reduction or prevention of use, the result can be the initiation (Strickland and Stoops, 2018) or continuation (Becker-Olsen and Briones, 2009) of illicit drug use. As these damaging consequences are borne from the PSA, they are considered direct harm. The unintended effects categorized under this heading include normalization of illicit drug use behaviour, increased interest in and use of illicit drugs and lack of PSA credibility. Stigmatization and marginalization are also very much aligned with this category, however, as these harms span multiple categories, they will be discussed separately.

#### Normalization and increased interest and use

While only a minority of participants in both cohorts acknowledged normalization and increased interest in and use of illicit drugs as unintended effects of PSAs, they recognized these outcomes as highly problematic and unacceptable. Portraying drug use, or its effects, could result in the normalization of drug use behaviour; that is, such a behaviour becomes acceptable as a norm and the perceived danger decreases (Erceg-Hurn, 2008). While illicit drug use prevention PSAs have the positive effect of raising awareness about the dangers of illicit drug use, this can result in piquing the interest among those at risk, potentially resulting in a desire to try or use drugs or accept the forbidden as an attraction or challenge (Crano *et al*., 2013; Strickland and Stoops, 2018). For example, while a small study conducted on an anti-methamphetamine use campaign resulted in 36.1% of participants wanting help to stop using, it was arguably overshadowed by 11.9% of the participants wanting to either *start* or *increase* methamphetamine use (Nanin *et al*., 2006).

#### Lack of credibility

The perspectives of practitioners from both sectors strongly concur with the evidence that governmental advertising lacking credibility can lead to ridicule from the target audience. Coupled with a general distrust in government, this leads to the overall dismissal of the message (Scheier and Grenard, 2010). When drug use consequences depicted in PSAs are perceived as exaggerated or lacking credibility, messages are often dismissed and rendered ineffective (Becker-Olsen and Briones, 2009; Cao and Xu, 2021). Historical research underscores this issue, showing that people who use drugs frequently reject governmental messages perceived as exaggerated or patronizing (Farrugia, 2014; Farrugia and Fraser, 2017; McEwen and Hanneman, 1972).

### Psychological, equity, group and social and opportunity cost harms

(Lorenc and Oliver, 2014) propose several examples of psychological harms, one of which is the generation of feelings of guilt, produced when prescribed behaviours are unable to be adopted (Guttman and Salmon, 2004). These feelings are psychologically damaging and can have negative effects on the behaviour targeted by the advertising campaign (Lorenc and Oliver, 2014). Similarly, illicit drug use prevention campaigns can use message appeals to invoke feelings of guilt, which can consequently exacerbate stigma in the target population, leading to further marginalization, less treatment-seeking and increased use of illicit substances (Meehan, 2017). Interventions targeted at specific groups or behaviours may contribute to further stigmatization (Lorenc and Oliver, 2014). This creates a barrier to the seeking of help for those using illicit drugs (Douglass *et al*., 2017; Lancaster *et al*., 2017). Additionally, if the stereotypical portrayals of those who use illicit substances are associated with ‘marginal status’ (Lorenc and Oliver, 2014, p. 289), the public health message will be rejected. Something not always considered as part of the unintended effects is the fact that funds spent on public communications, that provide seemingly little in the way of measurable effectiveness, could be redirected into more effective interventions (Lorenc and Oliver, 2014). In the illicit drug use space, this is a missed opportunity to direct these funds towards services such as rehabilitation, treatment, counselling, support.

#### Stigma and marginalization

Both groups of practitioners devoted most of their discussion time to the topics of stigma and marginalization. Initially, our interpretation was that these unintended effects were of equal importance to each sector. However, this proved not to be the case. Health support practitioners were adamant that stigma significantly contributes to creating and exacerbating barriers to seeking help for drug use and general healthcare. This phenomenon further marginalizes already vulnerable groups. They viewed stigma as a critical issue that impedes access to necessary support and services. Conversely, the advertising cohort considered stigma more as a challenge and a consideration, a perspective not widely discussed in the literature. One advertising participant mentioned avoiding fear and scare tactics, not due to concerns about stigma, but because these tactics are less effective when the audience is already stigmatized. Additionally, another participant from the advertising sector perceived the use of stigma as potentially beneficial in the context of illicit drug use prevention PSAs. These differing views can be attributed to the different lenses through which each cohort views the issue. The advertising cohort focuses on advertising effectiveness, such as whether the message reaches the intended target audience. In contrast, the health support cohort approaches the issue through a lens of behavioural change, emphasizing the broader social implications.

Stigma is arguably one of the most harmful effects of illicit drug use prevention PSAs. It encompasses four of the five harms identified by Lorenc and Oliver (2014)—direct, psychological, equity and group and social—often perpetuated through exaggerated and graphic portrayals of people who use drugs. Stigmatizing messages are *intended* to create fear and convey the social unacceptability of drug use (Lancaster *et al*., 2017; Marsh *et al*., 2017). However, such messages can have the opposite effect on minorities who already use illicit drugs, further isolating them and contributing to their social exclusion.

While the concept of opportunity cost harms wasn’t explicitly addressed, there were observations that health support professionals were keenly aware of the scarcity of treatment facilities. However, this concept was not mentioned by the advertising cohort. In the illicit drug use space, this is a missed opportunity to direct advertising funds towards services such as rehabilitation, treatment, counselling and other support services.

Although health support and advertising professionals operate in distinct sectors, both identified harms relevant to four of the five categories proposed by Lorenc and Oliver (2014)—direct, psychological, equity and group and social harms. This alignment between practitioners’ perspectives and the conceptual framework underscores the significance of applying theoretical models to understand the unintended consequences of illicit drug use prevention PSAs.

Four implications for practice for campaign development and implementation are drawn from this study. Firstly, to mitigate the most harmful risk identified, stigma, cross-disciplinary, multi-sector perspectives should be included and considered (Allara *et al*., 2015; Wakefield *et al*., 2010) as much as practicable and as early as possible in the process of campaign conceptualization. This will likely involve consultation with stakeholders from all relevant disciplines, including the two cohorts identified in this research, intended and unintended audiences, and those who use or have used illicit drugs. All perspectives should be considered, as the messaging might need to be fine-tuned to minimize possible negative effects on unintended audiences, i.e. depending on the medium used, there is difficulty ensuring only the intended audience sees the campaign, resulting in a spillover effect.

Secondly, during campaign development, it should be carefully considered who the unintended audience might be and what the potential effects might be, especially surrounding stigma (Douglass *et al*., 2017; Stead *et al*., 2019). During media planning, care should be taken to minimize the unintended audience receiving the message if that message could be potentially harmful to them or alter the message to minimize the potential harm.

Thirdly, conducting thorough pretesting is a crucial process to be undertaken prior to the release of any illicit drug use prevention campaign material. Pretesting can provide indications of things such as: whether the campaign will be understood and interpreted correctly by the intended audience, the identification of any potentially distracting elements or errors, and advice if any messages are offensive (Weinreich, 2010). Consideration should be given to extending this pretesting to include a broader range of stakeholders such as police, other support services, and the likely unintended audiences who might be harmed by the campaign.

The fourth implication involves improving integration between the campaign development team and other support services in the community to ensure the campaign does not deliver the message in isolation (Wakefield *et al*., 2010). An illicit drug use prevention PSA will have further reach than just the designated target audience. As the unintended audience and wider population may include individuals who are already using illicit substances, consideration should be given to running a complementary campaign for this population around how to access more support.

Naturally, there are limitations to be taken into consideration. Firstly, both advertising and commissioning agencies have documents, such as risk matrices and detailed instructions, they are unable to share for document analysis due to commercial-in-confidence status, which would have been informative to analyse. To better understand the commissioning decisions that impact on included content of illicit drug prevention PSAs, we suggest that future research is conducted to include perspectives of practitioners who are responsible for commissioning campaigns and creating policy in this arena. These perspectives could: provide insights into details of these sectors’ contributions, add to the understanding of the driving forces, enable identification of campaign development processes that may be improved, and further address literature and knowledge gaps.

A further limitation surrounds the smaller sample size for this qualitative work. While quantitative surveys are an alternative which can address the issue of generalizability (as many respondents can complete a survey providing results potentially generalizable to a broader population), semi-structured interviews are able to elicit in-depth, rich information in a way that quantitative surveys cannot, which is necessary to answer the research questions (Bryman, 2016). Although data saturation for both cohorts was reached after only a relatively small number of interviews when the absence of new or additional themes was forthcoming, this allowed the themes to be discussed in more detail.

## CONCLUSION

Missing from the literature are the perspectives of health support and advertising practitioners on the unintended effects of illicit drug use prevention PSAs in health promotion advertising. Interviews were conducted with cohorts from these two industry sectors and their perspectives have been analysed. Consolidated data from these sources suggest that practitioners are largely aware of the existence of unintended effects of illicit drug use prevention PSAs, yet their focus is often limited to their immediate task at hand. There is a lack of systemic understanding and purposeful consideration in the development of illicit drug use prevention PSAs that may be created at the campaign commissioning level.

This research has identified several practical recommendations for minimizing potential unintended campaign harms. As unintended harms are often borne from non-intended audiences receiving the campaign message, it is recommended that campaign pretesting includes broader populations to provide more opportunities to identify and mitigate the potential for harmful effects. Additionally, consultation with, and consideration of, the views of all stakeholders involved, not just in campaign development but also in wider campaign support, such as law enforcement, other support services, intended and unintended audiences is needed. Consideration should be given to running a complementary campaign for the population that is already using illicit drugs on how to access support.

## Supplementary Material

daae185_suppl_Supplementary_File

## Data Availability

The data underlying this article cannot be shared publicly for the privacy of the individuals who participated in the study.
