# Supplementary material for: Practitioners’ perspectives on unintended effects of illicit drug use prevention public service announcements in Australia
Source: Health Promot Int. 2024 Dec 6;39(6):daae185. doi: 10.1093/heapro/daae185 (PMC11630780; doi:10.1093/heapro/daae185)
Supplement: daae185_suppl_Supplementary_File [file daae185_suppl_supplementary_file.docx]

**Practitioners’ perspectives on unintended effects of illicit drug prevention public service announcements (PSAs) in Australia**

APPENDIX 1: INTERVIEW GUIDES

**Support Services Sector**

*Thinking about any advertising you have seen about illicit drug prevention, what do you think the goals and objectives of these campaigns are?

*What do you think are the intended consequences of these campaigns?

*What do you think they aim to do long term?

*Do you think there are any unintended consequences of these campaigns (these may be negative effects or positive effects of these campaigns)?

*If so, what are they, who may they affect?

*What might be a better or different approach?

*What strategies could be used to prevent any unintended effects of illicit drug use prevention campaigns?

*What tactics and messages used in these campaigns do you think should be avoided and/or emphasised? And why?

*What do you think about the effectiveness of public health campaigns, particularly those that aim to target illicit drug use?

*Who do you see as the most important audience/demographic that should be targeted by these campaigns?

*What are your feelings about the messages that these campaigns are trying to convey?

*Do you think there are any positive or negative consequences for the unintended audiences?

*What do you see as the biggest barriers to drug treatment seeking?

*What do you see that does encourage treatment seeking?

*Does your organisation commission any health campaigns?

If so, what sort of ‘brief’ do you provide (would you have an example you would be willing to share that will remain completely confidential? This will only be used for document analysis to gain a more in-depth understanding of the background rationale). This will be given full anonymity at an individual and professional level.

*Have you ever been consulted about the design/creation or pre-release promotion of public service announcements in the health or illicit drug use space? Including television media, posters, pamphlets, online etc

*If yes, what was the level of input etc

*If no, do you think your sector would be in a position to provide helpful insight into these campaigns? And what might this look like?

*Are you aware of the recommendations of the recent Parliamentary Joint Committee Report about public communications targeting drug and substance abuse?

*One is to implement a new campaign that will support law enforcement to reduce demand. It should also include shock and fear tactics to target the behaviour (rather than individuals) and be relatable.*

*While they recognise and acknowledge the potential to stigmatise (creating barriers to treatment seeking), their considerations are much broader than one segment of the population.*

*See stigma as an important societal behaviour-regulation tool.*

What are your thoughts on those rationales?

*Taking into account the committee recommendation, do you think that stigma could encourage those who do take drugs to seek treatment?

*Do you have any further thoughts about anything we have discussed today that I may not have covered?

**Advertising Sector**

*Thinking about the most recent illicit drug prevention or behaviour change campaign you worked with, can you describe it?

- what were the typical goals and objectives of the campaign?
- what was the call to action?

*Who, in the way of stakeholders, was consulted or involved in the design of this campaign?

*Who was the body/organization who commissioned and/or funded that campaign?

*What type of information did you receive (from the commissioner of the campaign) before conceptualizing the campaign?

*Do you have any examples of briefs or instructions (or a template) you have received that you would be willing to share (for document analysis only to gain a full understanding of the background rationale)? This will be given full anonymity at an individual and professional level.

*Was any pre-testing undertaken to ascertain any spillover effects this might have on certain demographics?

i.e., users vs non-users, adolescents vs adults, unintended audiences

*What was the target audience of the campaign?

*What were the main tactics and message appeals used (and/or avoided) in this campaign and why?

*What effects that may be unintended are considered?

*Are you aware of any evaluation/measurement that is produced post-campaign for effectiveness?

*Drawing on this conversation regarding consequences or effects, have you worked on any other illicit drug prevention campaigns?

*Are you aware of the recommendations of the recent Parliamentary Joint Committee Report about public communications targeting drug and substance abuse?

*One is to implement a new campaign that will support law enforcement to reduce demand. It should also include shock and fear tactics to target the behaviour (rather than individuals) and be relatable.*

*While they recognise and acknowledge the potential to stigmatise (creating barriers to treatment seeking), they see stigma as an important societal behaviour-regulation tool.*

*What are your thoughts on those rationales?

*Given some of the rationales behind the recommendation, would you consider using fear and shock-based stigmatising tactics or would it be something you would avoid?

*Has this conversation stimulated any other relevant ideas that I may not have covered?
